# National Action Plans for Antimicrobial Resistance in the WHO Western Pacific Region: The Just Transitions Lens

**DOI:** 10.1017/pub.2025.10097

**Published:** 2026-01-09

**Authors:** Ashish Giri, Sonia Lewycka

**Affiliations:** 1 Nuffield Department of Medicine, University of Oxford, Oxford, UK; 2 Oxford University Clinical Research Unit, Hanoi, Vietnam; 3 Centre for Tropical Medicine and Global Health, University of Oxford, Oxford, UK

**Keywords:** antimicrobial resistance, equity, Just Transitions, justice, National Action Plan

## Abstract

Antimicrobial resistance is one of the biggest global health threats, demanding urgent action. However, the threat of AMR and the ability to respond to it are far from evenly distributed. In many parts of the global South, where infectious diseases are more common, healthcare systems are weaker, and the social conditions that shape health are more precarious, the effects of AMR are felt more acutely. Because AMR arises within these unequal conditions, rapid action alone is insufficient; attention to how costs and benefits are distributed, who is prioritised, and whose voices are heard is essential to ensure that efforts do not inadvertently deepen existing inequalities. Hence, as the world prepares to tackle this “complex wicked problem,” it needs to ensure that we not only make a quick transition but also a just one. The first step in ensuring a just AMR transition is examining the fairness of our current strategies. This study critically reviews the National Action Plans of the countries in the WHO Western Pacific Region to examine the extent to which ideas of justice are considered. Findings reveal that there are limited considerations for how the burden and benefits of actions are shared; decision-making is dominated by external and national actors within human health. Access to antibiotics is restricted across the spectrum without adequately expanding and strengthening alternatives. As NAPs undergo revision, there is a need to more consciously and explicitly integrate considerations of justice and equity to avoid unintended harm and ensure effective outcomes.

Antimicrobial resistance (AMR) is a natural mechanism microbes use to evolve and protect themselves from threats.^1^ However, the overuse and inappropriate use of antimicrobials for human and animal health, and their leakage into the environment, accelerate this process and render critical drugs used to treat infections in humans, animals and plants ineffective.^2^ As a result, the treatment of infectious diseases becomes challenging in both humans and animals, leading to prolonged illness durations, extended hospital stays, and poorer treatment outcomes for both common illnesses and major surgical procedures.^3^

In the absence of any action, it is estimated that the impact of AMR will cost up to 3.8% of the global Gross Domestic Product (GDP), cause 10 million deaths, and push 28 million individuals into extreme poverty annually by 2050.^4^ Beyond the measurable toll of illness, death, and economic loss, AMR threatens food security and adversely impacts social and cultural aspects of life. With the consequences of inaction clear and already being felt, there is a need for urgent action to control AMR.^5^

## 1. The Global and National Action Plans for AMR

The current sets of global actions on AMR are guided by the Global Action Plan (GAP) that was developed by the World Health Organization (WHO) and adopted at the 68th World Health Assembly (WHA).^6^ The GAP serves as a blueprint and has been used to guide the development of National Action Plans (NAPs) in 178 countries.^7^ The NAPs outline each country’s vision and inform the nation’s response to AMR. Over the past ten years, countries have developed their NAPs and made varying but limited progress in implementing the planned actions and strategies.

## 2. The need for just actions

The need for urgent action on AMR is increasingly recognised. Yet, the need for these actions to also be just remains largely overlooked. Focusing on justice in this context is not only a moral imperative; it is also essential in ensuring that no one is left behind. Given the interconnected nature of AMR, prioritising the needs of people, geographies and sectors that are most affected by infections and AMR and have the least resources, power and capacity to respond strengthens both the effectiveness and fairness of our actions. Similarly, the inclusion of all stakeholders in decision-making allows addressing their concerns and navigating competing interests. Thereby making actions more socially and politically acceptable and more likely to be successful and sustainable. Finally, some actions aimed at mitigating the impact of AMR may have unintended negative consequences, and examining how the burden and benefits of our actions aimed at mitigating AMR are distributed helps in preventing some of these unexpected outcomes that could exacerbate existing inequalities or even give rise to new ones, potentially threatening community buy-in. Especially where the negative consequences of actions are most felt.^8^

The poor and vulnerable communities across the world face a higher burden of infectious diseases, are primarily agrarian, are more likely to have lower levels of education, and are extremely vulnerable to economic shocks.^9^ They also often reside in rural geographies that have limited access to doctors, diagnostics, and basic healthcare and have limited or no ability to pay for them.^10^ A combination of these factors, coupled with easy access to over-the-counter antimicrobials and insufficient knowledge of the appropriate use and potential harm of antimicrobials, leads to a heightened dependence on antimicrobials by these communities for both themselves and their livestock and crops.^11^ When policies seek to curb antimicrobial use without recognising these realities and without investing in measures that improve access to healthcare, they risk deepening existing inequalities and vulnerabilities.

The same is true for agriculture and farming. These sectors, particularly in the low- and middle-income countries (LMICs), have been under immense pressure to meet the global demand for affordable food and to secure profitable rural livelihoods. Adapting to this demand for producing more with even fewer resources, farmers, particularly in the LMICs, have adopted newer farming and agricultural practices that are characterised by high stock density, coexistence of farming, agriculture and aquaculture, increased human and animal contact, and prophylactic use of antimicrobials for growth promotion and disease prevention.^12^ Interestingly, these are also geographies that lack veterinary services and are severely deficient in animal healthcare infrastructure. Therefore, some of the much-needed and well-intended actions, if not thought through and implemented carefully, may potentially result in significant loss in yields, a drop in farmers’ income and national GDPs and threaten food security, especially for the poor.^13^

There is no question that AMR is a serious threat that demands bold and urgent action. However, actions that do not adequately take issues of justice into account may not achieve the desired impacts and may even give rise to unintended consequences.^14^ People with the highest burden of infectious diseases, the lowest ability to pay, and the least access to healthcare for themselves and their animals will end up experiencing most of the adverse unintended consequences and shouldering a disproportionate share of the responsibility for action without adequate resources and power.^15^ This may in turn further disrupt health and social well-being, exacerbate existing economic disparities, and engender multifaceted losses across various aspects of life.

## 3. A call for a “Just Transitions approach” in AMR

Therefore, as the world prepares to tackle this “complex wicked problem,” it needs to consciously make efforts to ensure that we not only make a quick transition to reduce antibiotic consumption and AMR but also a just one that equitably shares the benefits and costs, and responsibilities and power, and resources among all who are involved or affected.

The first step to ensuring a just AMR transition is to evaluate to what extent current global and national strategies and actions consider and take justice into account. This study does just that by critically examining National Action Plans in the WHO Western Pacific Region (WPR) using the Just Transitions lens.

The choice of the Just Transition lens was informed by the fact that the Just Transition approach has been used to approach similar challenges in the context of the labour movement and climate change with some degree of success.^16^ The approach emphasises treating all citizens equally in the process of planning development/change or transition and ensuring that the cost of transition is not disproportionately borne by vulnerable groups.^17^ While the approach is known, it gained significant prominence more recently when it was used to explore ways to reconcile actions aimed at limiting adverse climate impact with their unintended consequences, such as job losses, income losses, and the loss in taxes, in a manner that is quick, equitable, and just.^18^ A predicament very similar to the one we face with AMR.

However, this is not the only similarity between climate change and AMR that has encouraged the use of the Just Transition lens. Both climate change and AMR are global threats that transcend national boundaries.^19^ Second, both result from a complex interplay of multiple drivers making them “super wicked problems” that have no straightforward solution.^20^ Third, the utilisation of resources that contribute to climate change and the use of antimicrobials often prioritise individual benefits while neglecting the consequences for the broader community. Fourth, the impact of both is usually felt in the long term and is often diffused and relatively invisible at the point of use but permeates all aspects of life and disproportionately burdens impoverished and vulnerable communities, making the Just Transitions approach even more relevant.^21^

This study critically reviews the NAPs in the WPR through a Just Transition lens with an aim to evaluate the extent and context in which components of justice have been used. The study does not seek to evaluate or analyse the effectiveness of the NAPs but instead looks to find answers to the following questions:

1. What justice issues are considered/not considered and under what context?
2. What are the differences in justice issues considered across and within different strategic objective areas of the NAPs?
3. What are the opportunities to more consciously integrate Just Transitions into the NAPs?

The method used to answer these questions has been described in the Appendix.

## 4. Selection of NAPs

The WHO’s WPR has 37 countries, of which 26 had NAPs on AMR available on the WHO website. However, not all of them could be included in the review. Five NAPs were not published in English, one could not be downloaded, and the NAPs of Brunei and the Marshall Islands were available only in a picture format that did not allow key term searches. Five countries had two versions of the NAP, of which two had both NAPs in English. The most recent plans for China and Japan were not in English, and the one for Fiji was not downloadable. The old NAPs were included in these cases.

**Figure 1.**
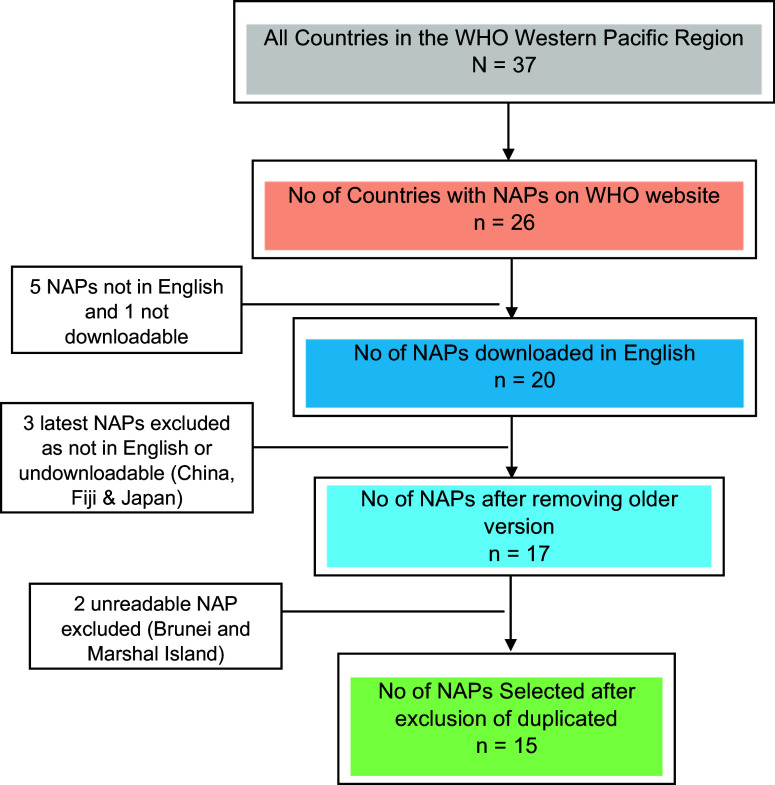
Schematic diagram of the selection of National Action Plans (NAPs).

After these exclusions, the review examined a total of 15 NAPs from 15 countries in the region. Of these, two NAPs were from high-income countries (HICs), five from upper middle-income countries (UMICs) and six from LMICs, as classified by the World Bank. Given these exclusions, the findings of the review are delimited to these 15 countries. Figure 1 schematically outlines the selection process and its result, while Table 1 classifies the selected countries of the WPR according to the World Bank classification of countries by income.^22^

**Table 1.** World Bank classification of countries by income

Country	Income group
Australia	High income
China	Upper middle income
Fiji	Upper middle income
Federated State of Micronesia	Lower middle income
Japan	High income
Cambodia	Lower middle income
Mongolia	Lower middle income
Malaysia	Upper middle income
Nauru	High income
The Philippines	Lower middle income
Papua New Guinea	Lower middle income
Tonga	Upper middle income
Tuvalu	Upper middle income
Vietnam	Lower middle income
Cook Islands	Not listed

## 5. The nature of the envisioned transition

The analysis of the overall vision and objective of the NAPs that outlines what the different countries in the WPR want to achieve in terms of AMR showed that only 9 out of the 15 NAPs stated a vision or overall goal. Visions and goals of only three out of the nine NAPs actually had wording related to social justice, equity, or fairness. Cambodia mentioned “social protection,” Micronesia mentioned “availability and accessibility of effective antimicrobials,” and Tonga mentioned making quality-assured medicines “accessible to all who need” them.

## 6. Tracing justice across the NAPs

The research used an identified set of key terms related to justice to extract sections from the NAPs to examine when the NAP considered aspects of justice and how did they do so. These terms were identified through a review of the four key WHO documents: the Global Action Plan on Antimicrobial Resistance (2016), the Agenda for Antimicrobial Resistance in the Western Pacific Region (2015), the WHO Six-Point Policy Package to Combat AMR (2011), and the WHO Global Strategy for the Containment of Antimicrobial Resistance (2001) using the Legal Geography “JUST” transition framework. This review resulted in the identification of 90 key terms related to justice. The largest number of words identified were under the distributive justice category *N* = 27, followed by recognition *N* = 19, Synonyms of justice *N* = 10, procedural justice *N* = 9, Restorative/reparative justice *N* = 6, Space *N* = 6, Time *N* = 6, and Cosmopolitanism *N* = 5.

**Figure 2.**
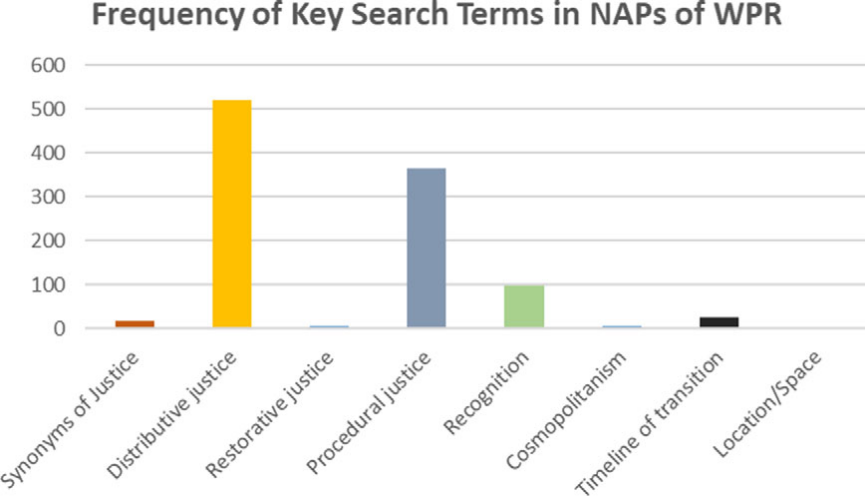
The category-wise frequency of terms in the selected National Action Plans (NAPs) in the region.

When the appearance of these terms was traced through the 15 available NAPs, an uneven pattern emerged. Terms linked to *distributive justice* appeared most frequently (519 times across 15 countries), followed by procedural justice (364 times) and recognition (98 times across 15 countries). By contrast, terms related to restorative justice, time, space, and cosmopolitanism had a low frequency of appearance (24 times across 7 countries, synonyms of justice—16 times across 9 countries, cosmopolitanism—6 across 4 countries, restorative justice—5 across 3 countries). Terms related to location/space did not appear in any of the NAPs. Table 2 and Figure 2 give details on the frequency of appearance of the terms in each category across the NAPs.

**Table 2.** The category-wise frequency of terms in the selected NAPs in the region

Classification	Search term thread	Frequency	Countries
Synonyms of justice	inequality or fair* or equit* or justice or equal* or injust* or unjust* or unfair* or unequit* or Just	16	9
Distributive justice	Distribut* or shar* or allocat* or Access* or avail* or affordab* or buy or pay*, Negative externalities or cost* or benefit* or gain* or loss* or impact* or public good or protect* or prescri*, Misus* or abus* or overus* or optimis*, use or optimiz*, appropriate, rational, irrational, indiscriminate	519	16
Restorative justice	Reparations or restor* (ative/ion/e) or repair* or correct* or undoing or redistribut*	5	3
Procedural justice	Procedur* or people centred, represent* or participat* or Partner* or collaborat* or consult* or involve* or multisectoral or engagement	364	16
Recognition	Social* or racial or rac*(ism/e) or class* or classism or cultural* or ecological* or intergenerational* or future generation*, gender* or patriarchy or ideological* or economic* or financial* or poor or poverty or income	98	15
	Marginalisation* or vulnerability* or hegemon*	0	NA
Cosmopolitanism	SDG* or health for all or sustainable development goals or universal healthcare or UHC	6	4
Timeline of transition	2030 or 2050 or 1998 or 2001, 2015, 2016	24	7
Location/Space	Developing country or developed country or LMICs or HICs or high income countr* or low and middle income countr* or emerging econom*	3	NA

*Note:* Figure 2 gives a schematic representation of the findings of this table.

Table 3 uses a heat map to show the distribution of terms by country visually. Table 4 uses a heat map to show the distribution of terms by category. In Tables 3 and 4, the greener the colour (colour online), the higher the frequency of the appearance of the key terms in the NAPs. The Philippines (22%, 21%, 50%), Japan (12%, 14%, 40%), and Cambodia (11%, 12%, 33%) accounted for most of the search term appearances related to distributive justice, procedural justice and recognition, respectively. While this analysis by itself has very limited inferential value, it serves as the groundwork for the next section, where an in-depth review of selected textual content from the NAPs of the region is undertaken to draw inferences and initiate discussions.

**Table 3.** Heat map depicting categories where most key terms across NAPs appeared

Country	Synonyms of justice *N* (%)	Distributive justice *N* (%)	Restorative justice *N* (%)	Procedural justice *N* (%)	Recognition *N* (%)	Cosmopolitanism *N* (%)	Timeline of transition *N* (%)	Location/Space *N* (%)	Total *N* (%)
Australia	2 (5)	15 (37)	0 (0)	15 (37)	9 (22)	0 (0)	0 (0)	0 (0)	41 (100)
Cambodia	3 (2)	55 (40)	0 (0)	42 (31)	32 (24)	0 (0)	2 (1)	2 (1)	136 (100)
China	0 (0)	8 (35)	1 (4)	7 (30)	7 (30)	0 (0)	0 (0)	0 (0)	23 (100)
Cook Islands	0 (0)	16 (43)	0 (0)	9 (24)	12 (32)	0 (0)	0 (0)	0 (0)	37 (100)
Fiji	1 (3)	13 (43)	0 (0)	11 (37)	5 (17)	0 (0)	0 (0)	0 (0)	30 (100)
Japan	2 (1)	61 (37)	3 (2)	52 (31)	39 (23)	2 (1)	7 (4)	0 (0)	166 (100)
Malaysia	2 (2)	41 (47)	0 (0)	29 (33)	14 (16)	0 (0)	2 (2)	0 (0)	88 (100)
Mongolia	1 (3)	13 (33)	1 (3)	11 (28)	12 (31)	0 (0)	0 (0)	1 (3)	39 (100)
Micronesia	0 (0)	33 (49)	0 (0)	16 (24)	18 (27)	0 (0)	0 (0)	0 (0)	67 (100)
Nauru	3 (5)	24 (43)	0 (0)	18 (32)	10 (18)	1 (2)	0 (0)	0 (0)	56 (100)
PNG	0 (0)	33 (44)	0 (0)	24 (32)	14 (19)	2 (3)	2 (3)	0 (0)	75 (100)
The Philippines	1 (0)	111 (46)	0 (0)	76 (31)	49 (20)	0 (0)	5 (2)	0 (0)	242 (100)
Tonga	0 (0)	29 (54)	0 (0)	16 (30)	8 (15)	0 (0)	1 (2)	0 (0)	54 (100)
Tuvalu	0 (0)	38 (48)	0 (0)	27 (34)	15 (19)	0 (0)	0 (0)	0 (0)	80 (100)
Vietnam	1 (2)	26 (44)	0 (0)	10 (17)	16 (27)	1 (2)	5 (8)	0 (0)	59 (100)

**Table 4.** Heat map depicting countries where key terms appeared most frequently

Country	Synonyms of justice *N* (%)	Distributive justice *N* (%)	Restorative justice *N* (%)	Procedural justice *N* (%)	Recognition *N* (%)	Cosmopolitanism *N* (%)	Timeline of transition *N* (%)	Location/ Space *N* (%)
Australia	2 (13)	15 (3)	0 (0)	15 (4)	9 (9)	0 (0)	0 (0)	0 (0)
Cambodia	3 (19)	55 (11)	0 (0)	42 (12)	32 (33)	0 (0)	2 (8)	2 (67)
China	0 (0)	8 (2)	1 (20)	7 (2)	7 (7)	0 (0)	0 (0)	0 (0)
Cook Islands	0 (0)	16 (3)	0 (0)	9 (2)	12 (12)	0 (0)	0 (0)	0 (0)
Fiji	1 (6)	13 (3)	0 (0)	11 (3)	5 (5)	0 (0)	0 (0)	0 (0)
Japan	2 (13)	61 (12)	3 (60)	52 (14)	39 (40)	2 (33)	7 (29)	0 (0)
Malaysia	2 (13)	41 (8)	0 (0)	29 (8)	14 (14)	0 (0)	2 (8)	0 (0)
Mongolia	1 (6)	13 (3)	1 (20)	11 (3)	12 (12)	0 (0)	0 (0)	1 (33)
Micronesia	0 (0)	33 (6)	0 (0)	16 (4)	18 (18)	0 (0)	0 (0)	0 (0)
Nauru	3	24 (5)	0 (0)	18 (5)	10 (10)	1 (17)	0	0 (0)
PNG	0 (0)	33 (6)	0 (0)	24 (7)	14 (14)	2 (33)	2 (8)	0 (0)
The Philippines	1	111 (21)	0 (0)	76 (21)	49 (50)	0 (0)	5 (21)	0 (0)
Tonga	0 (0)	29 (6)	0 (0)	16 (4)	8 (8)	0 (0)	1 (4)	0 (0)
Tuvalu	0 (0)	38 (7)	0 (0)	27 (7)	15 (15)	0 (0)	0 (0)	0 (0)
Vietnam	1	26 (5)	0 (0)	10 (3)	16 (16)	1 (17)	5 (21)	0 (0)
Total	16 (100)	519 (100)	5 (100)	364 (100)	98 (100)	6 (100)	24 (100)	3 (100)

## 7. Distributive justice: Distribution of the blame, resources, and the power to act

Distributive justice refers to “the equitable allocation of and access to material costs and benefits for all social groups in both spatial and temporal terms.”^23^ In the context of AMR, ensuring distributive justice would entail ensuring an equitable distribution of the blame for AMR and the distribution of power, responsibility, resources and actions to mitigate it across different sectors, regions and population groups.

### 7.1. Distribution of the blame

The “overuse”, “misuse,” and “irrational use” of antimicrobials in the human sector, followed by the animal and agriculture sector, were the most cited reasons for AMR across the NAPs.^24^ The articulation of this in the Malaysian and Mongolian NAPs, as an example, is as follows:

The indiscriminate use of antibiotics in human medicine and agriculture has driven the distribution of antibiotic-resistant bacteria.

Malaysian NAP

Doctors do not take attention on laboratory validation and do not have an interest to send patients for testing.

Mongolian NAP

Doctors usually have poor justification for antibiotic prescribing and there is a lack of control on prescribing, dispensing per prescription at pharmacy and use of antibiotics.

Mongolian NAP

Some countries also extended the blame to the inappropriate use of antimicrobials in aquaculture but acknowledged the need for further investigation.^25^ Australia and Fiji deviated from this and acknowledged that resistance occurs naturally and is hastened by inappropriate use. In contrast, China extended the blame for the emergence of AMR to emissions in the pharmaceutical industry and a lack of investment in new drug development. Fiji was the only country that mentioned the unavailability of antimicrobials as one of the factors contributing to AMR. It said:

Gaps identified in the procurement and supply of essential medicines, with stock outs reported leading to the unavailability of first and second-line antibiotics. This often resulted in inappropriate prescribing of third or last resort antibiotics.

Fiji

This “overuse,” “misuse”, and “irrational” use appear to be blamed on the “ignorance of the community,” weak guidelines, and poor surveillance systems.^26^ There were some references to the role of weak healthcare delivery systems in driving AMR, but these do not appear to be considered in planned actions.^27^ For example, the NAP for Tuvalu mentioned that it has only one hospital, and reaching this hospital is costly and may take as long as 36 hours. Despite this, activities under the responsible use of antimicrobials predominantly focused on limiting access through prescription-only antibiotics/antimicrobials, guidelines and consumption monitoring and did not mention any actions to improve access to hospitals/health facilities.

### 7.2. Distribution of the burden and costs of AMR

The need for urgent action across NAPs was most commonly emphasised by citing the burden of AMR, its economic cost and its implications on diseases and deaths. The data used to do so was often borrowed from global estimates.^28^ Where country-specific data on the burden of AMR were mentioned, they were limited to resistance in a small set of microorganisms.^29^ In the case of HICs like Australia, Japan, and to some extent, China, AMR was also said to have social and ecological costs and was mentioned as a biosecurity threat. However, none of the NAPs mentioned the distribution of these costs across social, demographic, and economic groups. Further, while calling for action, none of the NAPs considered the potential intended and unintended impact of these actions aimed at controlling AMR.^30^

### 7.3. The distribution of actions and resources to combat AMR

The stated objectives under which the proposed actions across the NAPs were mentioned included creating or enhancing AMR governance, promoting “rational/appropriate use” of antimicrobials, creating awareness, conducting surveillance and research, and promoting infection prevention.

The most common activities aimed at promoting “appropriate/rational use” of antimicrobials (antibiotics) appeared to be of two types. The first included the imposition of prescription-only antibiotics/antimicrobials, conducting audits and checks, and developing guidelines.^31^ The second type of activities promoting appropriate use were training, stewardship, quality control, and supply chain strengthening.

The Philippines, China, Japan, and Malaysia mentioned activities that go beyond the above-listed activities. The Philippines and Malaysia mentioned preferential market access to AMR-certified food and agricultural produce to promote appropriate use, while Japan proposed the development of a list of antimicrobials along with details of dosage, strengthening of surveillance and training of farmers and aquaculturists on appropriate antimicrobial use in the sector. China was the only country that looked beyond biomedicine and mentioned mainstreaming of traditional medicine as an action to improve access to healthcare and control AMR. Further, it also proposed a “new socialism countryside project” aimed at promoting broader systems strengthening in order to reduce the unnecessary use of antimicrobials. It said:

Combine the rational use of antibacterial agents with construction of a new socialism countryside project, bring scientific and literacy knowledge and medical services to rural areas, and integrate other policies of supporting and benefiting agriculture, rural areas and farmers to remove the unnecessary antimicrobials use.

NAP—China

Across the NAPs, infection prevention activities were most commonly centred on infrastructure development, the creation of standards and protocols, and the establishment of surveillance systems.^32^ However, these actions largely focused on human health, particularly on tertiary care inpatient facilities.^33^ Only a few countries—Australia, Japan, China, and the Philippines—focused on lower levels of healthcare provisioning. Six out of 15 NAPs mentioned implementation or monitoring of WASH practices as an infection prevention activity, out of which only Tonga, Micronesia, Tuvalu, and Fiji targeted these activities at the community level.

In the animal and agriculture sectors, too, the most common infection prevention activities were the development of standards, creation of awareness and training. As exceptions, Papua New Guinea, Malaysia, the Philippines, Fiji, Micronesia and Tonga, Japan, Australia, and made mention of the development or implementation of an “infection prevention and control program” or activities in agricultural, animal farms, and/or aquaculture. Tuvalu, Micronesia, Fiji, Japan, Malaysia, and Tonga mentioned strengthening vaccination in the animal and human sectors as a measure to prevent infection. The Philippines was the only country that mentioned strengthening the animal health system as a measure to prevent infection.

AMR in food production and food processing appeared to be an important focus area, with 10 out of 15 NAPs making mention.^34^ The most common actions in the area included the development of or compliance with standards, strengthening surveillance and testing. Japan and the Federated States of Micronesia mentioned the promotion of good hygiene practices and hazard analysis at critical control points.

There were rarely dedicated actions that were targeted at the environmental sector. When mentioned, the actions primarily focused on appropriate waste management in hospitals and disposal of outdated antimicrobials through the development of guidelines, inspections or strengthening of existing protocols.^35^ Fiji and China were exceptions as they mentioned the development of legislation to promote environmental health. Fiji mentioned waste management across different sectors as a dedicated focus area, while China listed improving surveillance to regulate antimicrobial environmental pollution in water and soil, emission reduction of antimicrobial agent waste, development of a system to evaluate antimicrobial-caused environmental hazard and the need for environmental impact assessment for site selection/expansion of pharmaceutical companies.

Training and awareness were common to both infection prevention and the appropriate use of antimicrobials.^36^ However, training appeared to focus more on professionals in human health compared to animal health and aquaculture and again mostly targeted tertiary hospitals.^37^

The content of training was related to standards, guidelines and best practices and often went along with investments in infrastructure strengthening, purchase of equipment and additional dedicated human resources.^38^ Awareness comprised developing and sharing materials with the communities, policymakers or farmers and observing AMR awareness week.^39^ A few countries—Australia, Tonga, Nauru, Japan, and the Philippines differed in targeting farmers, communities and policymakers with a more structured and sustained approach to improving understanding related to AMR through monthly sessions and engagement. Japan also expanded training to include people in the local government leadership.

For research activities, HICs—Japan, Australia, and China—had more comprehensive and structured research priorities. They appear to focus more on the development of new antimicrobials, technologies, vaccines, genomics, and infection prevention, mostly in the context of human health. Japan and China are exceptions in prioritising similar research beyond human health and have the following activities mentioned:

Development and usage of vaccines for livestock, farm-raised aquatic animals and pets.

NAP Japan

Controlling environment contamination caused by antibacterial agents.

NAP China

LMICs and UMICs, in contrast, appeared to lack a clear or detailed research agenda.^40^ The Cook Islands did not have any reference to research at all. While Papua New Guinea, Cambodia, Tuvalu, and Fiji mentioned research, they appeared to focus on measuring the burden of AMR, estimating its impact and developing standard operating procedures. Vietnam and the Philippines stood out in having a more detailed and clear research agenda that included the development of vaccines and diseases of viral origin. The focus of research in these two countries also went beyond measuring the disease burden or benefiting human health in tertiary care settings. The NAP of the Philippines said:

Prioritising research that benefits small-hold farmers (i.e. concepts and technology they could use in their farms).

NAP Philippines

Finally, while most NAPs recognised the threat posed by infections of fungal, viral origin and Malaria, the focus of most actions appeared to be on diseases of bacterial origin (TB excluded). This was evident from the fact that most NAPs used the term “antimicrobials” in the background and sections that highlighted the challenge or even when listing the objective of actions but used the term “antibiotic” in describing the activity itself. While most countries did not mention TB, HIV and Malaria, Papua New Guinea, Mongolia, Micronesia, Cambodia, and Tuvalu recognised the growing threat they pose, only Vietnam and Nauru dedicate actions to address them.

## 8. Procedural justice

Procedural justice refers to ensuring that all stakeholders are heard, included and involved in decision-making processes.^41^ In this section, we try to explore the different actors and their involvement in the development, governance, and implementation of the AMR agenda in countries in WPR.

### 8.1. Participation in the planning/development of NAPs

The process of development of NAPs in most LMICs and UMICs started with a situation analysis and is followed by one or more multi-stakeholder workshops.^42^ The situation analyses and the multi-stakeholder workshops were either led by or supported by international organisations such as FAO and WHO.^43^ The NAPs of HICs made no reference to such analysis or stakeholder workshops. They instead mentioned their national surveillance systems or their previous NAP and appeared to use existing data to guide their NAPs.^44^

The workshop participation varied across countries but most often was limited to representation from the government sector and the WHO. Most commonly, the national ministries of animal and human health, environment, agricultural, and food sectors (or their equivalents) were mentioned, but without further details. There was no mention of community, civil society, academia, professional unions, and so forth in these workshops that led to the creation of NAPs.

### 8.2. Participation in AMR governance

In most countries, the AMR agenda was governed by a national body that was always headed by the Ministry of Health.^45^ The composition of the committee was mostly limited to the government ministries, except for in the Cook Islands, Fiji, Papua New Guinea, and Nauru, where the civil society, professional bodies, and private sector were also represented.

This committee in Papua New Guinea, the Federated States of Micronesia, the Philippines, Nauru, and Australia was complemented by one or more subordinate committees and was responsible for providing implementation or advisory oversight. These subordinate committees had wider representation, including civil society and academia, but the apex body continued to operate only at the national level, except for Micronesia, which also had it at the state level.

Vietnam, Cambodia, Japan, the Philippines, and China did not outline the governance structure of AMR. However, Japan and the Philippines listed important stakeholders for every listed objective of the NAP. The stakeholders in the case of the Philippines were the most diverse and the NAP highlighted the need to further strengthen it.

### 8.3. Partnerships for implementation

The recognition of the need for collaboration and partnership to mitigate challenges posed by AMR was clearly articulated across all the NAPs. In line with this recognition, NAPs mention four distinct types of partnerships.

The first type of partnership was between the different ministries. The focus of this type of partnership was on the exchange of data that mostly flowed from the bottom to the top or across ministries and was of national character.^46^ The Philippines stood as an exception and mentions sharing information from the analysis of data back to the farmers. Further, the Philippines, along with Japan, mentioned engaging with lower levels of governance for training, surveillance and stewardship.

The second type of partnership was that between the government and the community, civil society, and private organisations. The nature of these partnerships appeared to be very unequal, with the role of civil society limited to the creation of awareness and that of the community to a passive receiver of information.

The third type of partnership was the one involving international organisations. These organisations were listed as important partners in the NAPs of the LMICs and UMICs. They appeared to play a critical role in conducting situation analysis, have a seat/influence in the AMR governance structure and are mentioned as a source of funds to finance AMR activities.^47^

The final type of partnership was between countries. However, there appeared to be a significant difference between what HICs and LMICs focused on through these partnerships. While the NAPs of LMICs and UMICs mentioned such international partnerships in the context of participation in global workshops, receiving training and sharing data.^48^ HICS like Japan and Australia appeared to view these partnerships and collaborations as a way to emerge as a global leader and as an opportunity to influence “the AMR agenda in the region.” For example, Japan, in the background section of its NAP mentioned:

Japan has been tackling AMR issues since the 1990s and this experience puts Japan in the position to take leadership for the world, especially for Asian-Pacific countries.

NAP Japan

While Australia had the following listed as a priority action area in its NAP.

Influence [ing] the global antimicrobial resistance agenda by active engagement and collaboration with other countries, multilateral organisations and forums.

NAP Australia

Consequently, the international partnerships originating in HICs and China had distinct activities. These included the creation of standards and guidelines, data sharing and research, capacity building and training, and donations and aid and convergence with enterprises and international bodies like GLOPID-R (Global Research Collaboration for Infectious Disease Preparedness) and the G20 (Group of Twenty, an intergovernmental forum of major economies).

## 9. Restorative justice

Restorative justice has to do with recognising historical injustice in the planning and design of current action.^49^ The NAPs appeared to be deficient in their considerations of restorative justice. There was no acknowledgement of how certain geographies and population groups may have been historically disadvantaged in terms of the burden of diseases, access to healthcare or the economy.^50^

Consequently, there were no actions aimed at restorative justice, either in terms of correcting the existing inequalities or minimising the potential for new ones that may arise from current actions. There was one example of restorative justice in the NAP of the Philippines, where covering the cost of hospital-acquired AMR in patients through insurance is proposed.

## 10. Recognition of the differential capacities and need for action

The actions listed in the NAPs across the countries appear to have a limited recognition of the differential burden of diseases across social, economic, demographic, and geographic population groups.

The NAPs did appear to recognise the differential capacities and needs in terms of the existing understanding of the burden of AMR, strength of surveillance systems, awareness, skills and resources, especially between the human, animal, and aquaculture sectors.^51^ The Australian NAP acknowledged these differences accurately and said:

Antimicrobial stewardship cannot be a ‘one size fits all’ solution.

Australia

However, recognition of differential capacities and needs did not always translate into differential or prioritised action.^52^ For example, despite recognition of the threat of AMR in the animal sector and aquaculture, most countries continued to focus on human health.

## 11. Cosmopolitanism: Alignment with global goals

Cosmopolitanism refers to the recognition of an issue’s global nature and interconnectedness. In the context of AMR, they refer to how they relate to or are influenced by the global goals. Most NAPs did not mention common global goals like the Sustainable Development Goals (SDGs) or Universal Health Coverage (UHC). The only exceptions were Japan, Nauru and Papua New Guinea, which mentioned the terms in their background detailing the threat AMR poses to SDG and UHC or their current poor performance in terms of achieving UHC.

However, the analysis of the overall vision and objective of the NAPs shows that NAPs closely aligned with and appeared to be influenced by the Global Action Plan and other WHO-published regional documents on AMR. The articulation and focus of the stated overall vision or goals of the different NAPs appeared to be influenced by the documents the NAPs make mention of.

For example, the NAP of Papua New Guinea made reference to the GAP, and the articulation of its vision/goal aligns closely with the way the overall vision of the GAP is articulated.

The overall goal of the action plan is to ensure, for *as long as possible*, continuity of the ability to *treat and prevent infectious diseases with effective and safe medicines* that are quality-assured, used in a responsible way, and accessible to all who need them.

Stated overall goal of Global Action Plan

A country that continues to *benefit from the use of effective antimicrobials* for the treatment of infections in humans and animals for a *long time.*

Stated Vision in the NAP of Papua New Guinea

In contrast, the Philippines did not make reference to global documents but instead made references to its previous NAP.^53^ It mentioned that “the current plan (in the current NAP) maintains the key strategies from the original NAP (old NAP)” and articulates the overall vision for the current NAP.

A nation *protected against the threats* of antimicrobial resistance.

Stated Vision of NAP of Philippines

The Cambodian NAP attributed the current progress in the area of AMR in the country to three different documents, saying:

Cambodia’s recent achievements on antimicrobial resistance were anchored in the AMR Country Situation Analysis Report 2013, the National Policy to Combat Antimicrobial Resistance in Cambodia (2014) and the National Strategy to Combat Antimicrobial Resistance 2015–2017.

NAP Cambodia

In the subsequent section of the NAP, it defined its overall goal as:

The Multi‐Sectoral Action Plan Visualises *a country with a healthy population and strong governance systems* to control the threat of AMR that would impede economic growth and cause unnecessary risks to health, security and social protection.

Stated Overall Goal of NAP of Cambodia

Nine of the 15 NAPs stated a vision or overall goal. The vision, along with the document NAPs make reference to is shown in Table 5.

**Table 5.** Stated vision of NAPs and common documents referred

Country	Stated vison/Overall goal of NAP	Document referred
Cambodia	The Multi-Sectoral Action Plan visualizes a *country with a healthy population* and strong governance systems to control the threat of AMR that would impede economic growth and cause unnecessary risks to health, security and social protection	1. Country Situation Analysis Report 2013 2. National Policy to Combat AMR in Cambodia (2014) 3. National Strategy to Combat AMR 2015–2017
Japan	To *minimize the emergence of AMR* and prevent the spread of infectious diseases caused by antimicrobial-resistant organisms towards a *world free of the burden of infectious diseases* caused by AMR	Mentions past works, does not mention a document
Malaysia	To slow the *emergence of AMR* and prevent its spread	Not mentioned
Mongolia	The goal of the National Action Plan is to *promote rational use of antibiotics* & prevention of the emergence and spread of AMR, improve surveillance of AMR & diagnostics, treatment of antimicrobial infections, and enhance quality of hospital care & outcomes	GAP
Micronesia	To maintain a healthy population in the Federated States of Micronesia by ensuring the *availability and accessibility of effective antimicrobials*	1. Action Agenda for AMR in the Western Pacific Region 2. GAP
Nauru	To prevent, control and *minimise the impact of AMR* in Nauru	Action Agenda for AMR in the Western Pacific Region
Papua New Guinea	A country that continues to benefit from the use of *effective antimicrobials* for the treatment of infections in humans and animals for a long time	1. Country Situation Analysis *on AMR (conducted with support of WHO)* *2. GAP*
The Philippines	A nation protected against the *threats of antimicrobial resistance*	Previous NAP
Tonga	To ensure, for as long as possible, continuity of *successful treatment and prevention of infectious diseases* with effective and safe medicines that are quality-assured, used in a responsible way, and accessible to all who need.	Action Agenda for AMR in the Western Pacific Region

*Note:* Australia, China, Cook Islands, Fiji, Tuvalu, and Vietnam do not mention an overall goal or vision.

## 12. Time and space

The NAPs are national plans, and therefore, the focus of actions and vision of NAPs of most countries is limited by the national boundaries of the country. However, HICs like Australia, Japan, and China list activities related to research, surveillance, and data sharing that transcend national boundaries.

While most NAPs have a period for which they are applicable, there is no timeline for achieving the transition or the implementation of activities. Vietnam is an exception with clear timelines for each action.

## 13. Discussion

### 13.1. Actors setting and driving the AMR agenda

Our analysis of the NAPs in the WPR shows that the international organisations and UN bodies like FAO and WHO have played a significant role in the development of NAPs, particularly in the case of LMICs and UMICs and were also present in the national AMR governance machinery, offering technical and advisory support. National actors, in turn, often viewed these external partners as important sources of funding for the AMR agenda, frequently naming them as key funding sources across the NAPs.^54^

Like other areas of global health governance, AMR policy and governance are subject to the influence of power asymmetries.^55^ Therefore, without adequate consideration and efforts to incorporate the voices of diverse stakeholders, especially the marginalised, there is potential for influential organisations’ structural, epistemological, and normative preferences to disproportionately influence the problematisation of the issue, priority setting, action selection, and distribution of resources.

For instance, the NAPs of Japan and Australia, HICs in the WPR list, “influencing AMR agenda in the WPR” as one of their objectives, and “building capacities” in other countries of the region through development assistance as an activity. Health diplomacy and developmental assistance are known tools for international relations and are key to assuming coveted positions in the global order.^56^

Therefore, it is important to consider how HICs engage with LMICs. In the absence of adequate foresight and care about the existing inequalities and the unintended consequences of their actions, there is a possibility that even the well-intentioned aid and assistance from HICs will impact what is prioritised and who is neglected, making the AMR agenda more externally than internally driven. Thereby, making the AMR agenda more externally than internally driven. The outcome of this could be detrimental not just for the equitable distribution of costs, responsibility of action and resources but also for the ownership and sustainability of the transition.

### 13.2. Evidence and disparities informing current actions

In relation to the evidence and rationale for current actions, our results show that most NAPs, in LMICs, do not mention the evidence that quantifies the burden of AMR and its impact in their country. On the contrary, NAPs of the Philippines, Papua New Guinea, and Tuvalu explicitly acknowledge the lack of such evidence. When data is mentioned, they are estimates and are usually derived from global estimates of the impact of AMR.^57^ When country estimates were provided, they usually focused on resistance in a few common diseases and specific microbes in human health.^58^

In contrast to this scarce context-specific data in relation to AMR in LMICs, there is enough evidence that highlights the differential burden of diseases, access to healthcare and the impact of these diseases beyond financial losses across different population groups both within the country and across the globe. Women, children and people living in LMICs, especially the poor, the illiterate and those living in rural geographies, are known to share a disproportionate burden and have the least access to healthcare.^59^ Despite this, there is no mention or recognition of it in the NAPs or in the actions that are proposed as strategies to control access to antimicrobials, prevent infections, undertake research or raise awareness in the community.

The burden of AMR and its potential impact cannot be generalised. It is crucial to consider existing political, economic, social, and epidemiological differences.^60^ Understanding the interplay of these elements is essential to grasp the true burden of AMR, identify its drivers, assess its impact, and explore potential effective solutions that share the costs and benefits of action and inaction equitably. Solutions that do not recognise the realities of the disadvantaged may simply not be enough to meet the needs of the most vulnerable and may adversely impact their health and wellbeing.

### 13.3. Perspectives and epistemologies informing current actions

Biomedicine was the dominant perspective that the current NAPs take to problematise and design solutions for AMR mitigation. The Chinese NAP is the only exception.

This is despite the fact that the countries in this region are known for traditional medicines, and in LMICs a significant proportion of the population living in rural geographies is dependent on alternative forms of medicines to treat both infectious and non-infectious diseases.^61^ Further, there is evidence that suggests that practices and treatments used in alternative forms of medicine are effective in treating infectious diseases and training in integrative medicine results in lower antibiotic prescriptions.^62^

Therefore, excluding alternate and traditional forms of medicine that are outside of biomedicine is not only epistemologically unjust but may also make some of the strategies irrelevant to people practising it and communities dependent on it for care.

AMR is a complex and multi-sectoral challenge, driven by interconnected human, animal, and environmental practices and shaped by structural inequities.^63^ Addressing it effectively and fairly therefore, requires a more pluralistic approach, one that brings together expertise from across disciplines and sectors and different ways of knowing, including community and traditional knowledge systems.^64^ Integrating such diverse perspectives is essential for developing AMR strategies that are not only scientifically sound but also socially relevant, equitable, and context-appropriate.

### 13.4. Responsibility for action versus concentration of power and resources

The success of the envisioned AMR transitions is largely dependent on the appropriate use of antimicrobials by communities. Especially by the farmers and aquaculturists, where over 70% of all antimicrobials are used.^65^ Despite this, all committees and AMR governance bodies were highly centralised and headed by the Ministry of Health, with the animal, agriculture and environment sectors often under-represented and occupying a subordinate position. Who is present in the committee and whose voices are valued determine what is prioritised and how resources and costs of action are distributed.

Without equitable representation and fair distribution of power and responsibilities across different stakeholders, there is a risk of pushing the burden of AMR action onto actors who lack the resources or authority to act effectively. For example, when communities are expected to implement infection prevention and control (IPC) measures without parallel investments in infrastructure, such as veterinary extension services, clean water, waste systems, or basic laboratory capacity, these efforts are likely to falter, eroding both equity and community trust.^66^

Most NAPs emphasise curbing “irrational use” through restrictive prescription policies, and infection prevention and stewardship initiatives are concentrated in human health and tertiary facilities. This, while benefiting communities living in cities where such facilities are mostly located, creates a dangerous asymmetry: communities and their animals living in geographies that are far from such facilities may face reduced antimicrobial access without corresponding reductions in infection risk. This may result in increased cost of seeking care and delayed care-seeking behaviour in humans while also risking acceleration of animal disease outbreaks, threatening livelihoods, and incentivising unregulated or informal antimicrobial use, undermining the very goals of stewardship.

Even when improving access to antimicrobials was mentioned, it was in the context of human health and through actions aimed at monitoring the quality of the drugs and strengthening the supply chain.^67^ Such activities are more likely to benefit people living close to functional health facilities and leave behind people who are dependent on animals and live further away.

Within human health, the listed activities in most NAPs do not have dedicated actions on diseases like TB, HIV, and malaria. In most countries, these diseases have their own vertical health programmes and, thereby, may be perceived to be less vulnerable to resistance. However, these diseases require immediate and often long-term access to medicines and disproportionately burden the poorest, who live in geographies that do not have access to doctors and diagnostics, and in such settings antiretroviral, antimalarial, and medicines for tuberculosis are rapidly becoming ineffective.^68^

## 14. Limitations

The search terms related to justice, fairness and equity were identified using the “JUST” legal geography framework by reviewing four WHO documents that guide policies and actions related to AMR. The Just Transition approach evolved from the climate change and labour movement. The considerations of justice, fairness, and equity in these movements might have manifested and been articulated differently from the way they are in the context of AMR, which could be a limitation. Further, fairness, equity, and justice are complex concepts, understood differently in different social and cultural settings and articulated in a variety of ways in different languages. The identified key terms in English may not capture these comprehensively. Consequently, by extracting sections where these terms appear in NAPs and analysing them, there is a possibility of missing considerations of justice, fairness, and equity that were made but articulated differently.

## 15. Strengthening Justice in AMR: Gaps and Opportunities

The analysis of NAPs in the Western Pacific Region underscores an urgent need to act on AMR. However, these plans either overlook or inconsistently address critical justice and equity considerations—failing to ask who bears the cost, who decides, and who benefits. They neglect the deep-rooted inequalities that shape how AMR is experienced and addressed, including disparities in disease burden, health system capacity, social determinants of health, and the ability to comply with mitigation strategies. Moreover, they often fail to anticipate the unintended consequences of AMR actions, which are likely to fall most heavily on the already vulnerable.

Despite AMR being a One Health challenge, the process of development of NAP and the AMR governance mechanism is centred around the Ministry of Health and influenced by external stakeholders. While a large component of behaviour change is expected at the level of the community, their involvement in planning and implementation is limited, and there are very few, if any, actions that enable them to make that change.

Actions like limiting access to antimicrobials are applied across sectors and communities, while most resources, infection control activities and training are targeted at human health, especially tertiary care. In contexts marked by structural inequalities, weak health and veterinary systems, and fragile livelihoods, actions that are not attuned to local realities risk deepening existing disparities and undermining the very communities most affected by AMR.

Nevertheless, some NAPs do show promising practices. Therefore, as the new GAP is introduced and countries begin revising their NAPs, the Just Transitions framework offers a timely and practical approach to embed considerations of justice, fairness, and equity, ensuring AMR strategies are not only technically effective but also socially inclusive, locally owned, and ethically grounded.

## Figures and Tables

**Figure A1.**
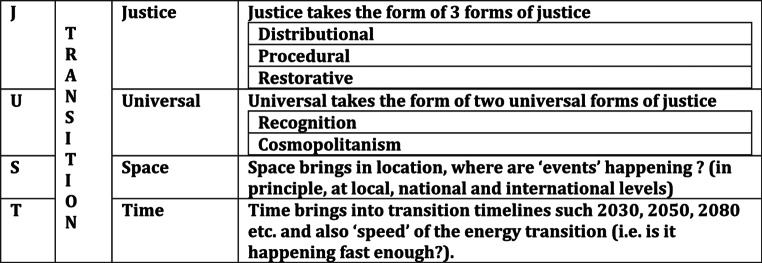
The legal geography “JUST” framework for a Just Transition.

**Figure A2.**
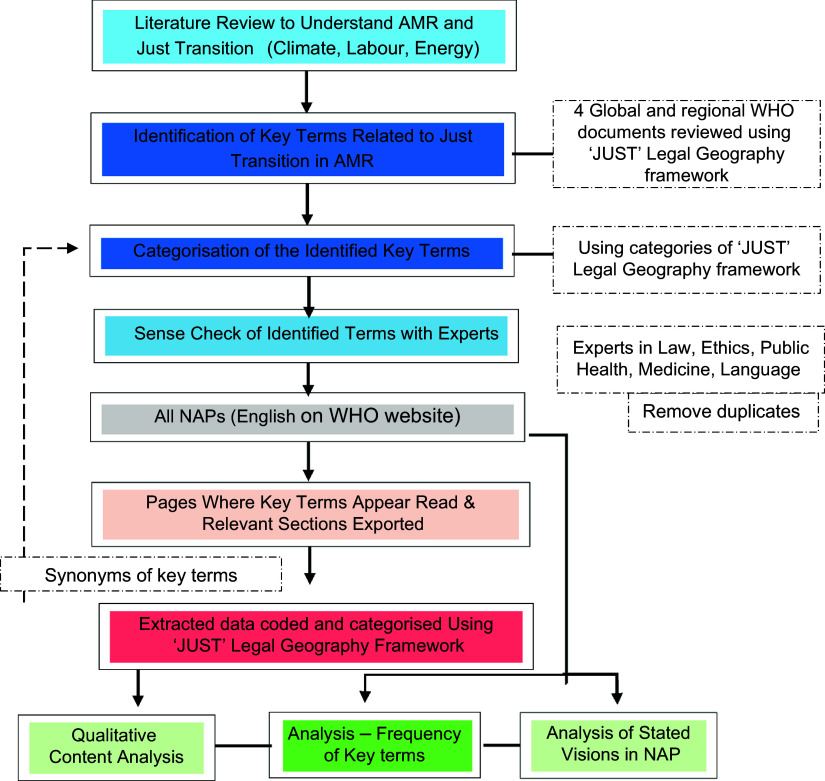
Schematic diagram of the methodology.

## Footnotes

1 Salam et al. 2023.

2 Haenssgen et al. 2021.

3 Dadgostar 2019.

4 O’Neill 2016.

5 Littmann, Viens, and Silva 2020; Parsonage et al. 2017.

6 Van Heuvel et al. 2022.

7 Quadripartite Joint Secretariat on AMR 2025.

8 Serrano 2022.

9 Bishwajit, Ide, and Ghosh 2014.

10 Strasser, Kam, and Regalado 2016.

11 Pokharel, Raut, and Adhikari 2019.

12 Ikhimiukor et al. 2022; Lam, Pham, and Nguyen-Viet 2018; Rohr et al. 2019.

13 Littmann and Viens 2015; Selgelid 2007.

14 Parsonage et al. 2017.

15 Littmann and Viens 2015; Parsonage et al. 2017; Selgelid 2007.

16 McCauley and Heffron 2018.

17 Pinker 2020.

18 McCauley and Heffron 2018; Wang and Lo 2021.

19 Harring and Krockow 2021.

20 Harring and Krockow 2021.

21 Anomaly 2013.

22 World Bank Open Data n.d..

23 Schlosberg 2013.

24 NAP—Papua New Guinea, Mongolia, Federated States of Micronesia, Tonga, Cambodia, Vietnam, Tuvalu, Cook Islands, Malaysia.

25 NAP—Philippines, Nauru, Japan, Vietnam, Malaysia.

26 NAP—Philippines, Republic of Nauru, Japan, Vietnam, Malaysia.

27 NAP—Papua New Guinea, Mongolia, Cambodia, Tuvalu, Fiji.

28 NAP—Philippines, Tuvalu, Malaysia, Australia.

29 NAP—Papua New Guinea, Philippines, Tuvalu.

30 NAP—Cook Islands, Federated States of Micronesia, Fiji, Malaysia, Mongolia, Cambodia, Papua New Guinea, Japan, Philippines, China, Nauru, Tuvalu, Australia, Tonga, Vietnam.

31 NAP—Papua New Guinea, Mongolia, Micronesia, Tonga, Nauru, Cambodia, Tuvalu, Cook Islands, Malaysia, Fiji.

32 NAP—Cook Islands, Federated State of Micronesia, Fiji, Malaysia, Mongolia, Cambodia, Papua New Guniea, Japan, Philippines, China, Nauru, Tuvalu, Australia, Tonga, Vietnam.

33 NAP—Papua New Guinea, Mongolia, Federated State of Micronesia, Tonga, Cambodia, Vietnam, Tuvalu, Cook Islands, Fiji, Malaysia.

34 NAP—Tuvalu, Cambodia, China, Federated State of Micronesia, Japan, Malaysia, Nauru, Philippines, Papua New Guinea, Tonga.

35 NAP—Cook Islands, Federated State of Micronesia, Nauru, Malaysia, Tonga, Cambodia, Papua New Guinea.

36 NAP—Papua New Guinea, Mongolia, Micronesia, Tonga, Philippines, Nauru, Cambodia, Japan, Vietnam, Tuvalu, Cook Islands, Malaysia, Australia, Fiji, China.

37 NAP—Papua New Guinea, Mongolia, Micronesia, Nauru, Cambodia, Vietnam, Tuvalu, Cook Islands, Malaysia, Fiji.

38 NAP—Papua New Guinea, Federated State of Micronesia, Mongolia, Nauru, Cambodia, Vietnam, Tuvalu, Cook Islands, Malaysia, Fiji.

39 NAP—Cook Islands, Federated State of Micronesia, Fiji, Malaysia, Mongolia, Cambodia, Papua New Guniea, Japan, Philippines, China, Nauru, Tuvaly, Australia, Tonga, Vietnam.

40 NAP—Papua New Guinea, Cambodia, Cook Islands, Malaysia, China.

41 Schlosberg 2013.

42 NAP—Papua New Guinea, Philippines, Nauru, Cambodia, Cook Islands.

43 NAP—Papua New Guinea, Philippines, Nauru, Cambodia, Cook Islands.

44 NAP—Japan, Malaysia, Australia, China.

45 NAP—Mongolia, Micronesia, Philippines, Nauru, Cook Islands, Malaysia, Australia, Fiji, China.

46 NAP—Papua New Guinea, Mongolia, Tonga, Philippines, Nauru, Cambodia, Japan, Vietnam, Cook Islands, Malaysia, Australia, Fiji.

47 NAP—Papua New Guinea, Mongolia, Micronesia, Tonga, Nauru, Cambodia, Vietnam, Tuvalu, Cook Islands, Fiji.

48 NAP—Papua New Guinea, Mongolia, Micronesia, Tonga, Cambodia, Tuvalu.

49 Schlosberg 2013.

50 NAP—All countries.

51 NAP—Papua New Guinea, Micronesia, Philippines, Cambodia, Japan, Tuvalu, Australia, China.

52 NAP—Papua New Guinea, Cambodia, Tuvalu, Cook Islands.

53 Philippines, Inter-Agency Committee on Antimicrobial Resistance 2019.

54 NAP—Papua New Guinea, Micronesia, Tonga, Nauru, Cambodia, Tuvalu, Cook Islands, Fiji.

55 Kickbusch and Liu 2022; McBride, Hawkes, and Buse 2019.

56 McBride, Hawkes, and Buse 2019; Rudolf 2022.

57 NAP—Cambodia, Vietnam, Malaysia.

58 NAP—Papua New Guinea, Mongolia, Tonga, Cambodia, Cook Islands, China.

59 Bhutta et al. 2014; Dunn and Nhlema 2005; GBD 2019 Child and Adolescent Communicable Disease Collaborators 2023; Selgelid 2007.

60 Dar et al. 2016; Pokharel, Raut, and Adhikari 2019.

61 Park and Canaway 2019.

62 Dar et al. 2016; Oladunjoye et al. 2022; van Wietmarschen 2020.

63 Bennett et al. 2024; WHO 2021.

64 Calvo-Villamañán, Millán, and Carrilero 2023; Cheah, Lewycka, and de Vries 2025.

65 Van Boeckel et al. 2017.

66 McCauley and Heffron 2018.

67 NAP—Papua New Guinea, Mongolia, Tonga, Nauru, Cambodia, Tuvalu, Cook Islands, Malaysia, Fiji.

68 Dunn and Nhlema 2005; Goldberg, Siliciano, and Jacobs 2012; WHO n.d.-b.

## Appendix: Methodology

This study uses the “JUST” framework developed in legal geography to critically review the National Action Plans (NAPs) in the Western Pacific Region.^69^ The JUST framework was chosen over alternative justice frameworks such as energy justice, environmental justice, and Rawlsian distributive justice because it offers a comprehensive, interdisciplinary, and spatially grounded lens. Drawing on insights from complex challenges like climate justice, energy transitions, and labour movements that share similarities with AMR, the JUST framework integrates distributional, participatory, and restorative justice, while also incorporating temporal and spatial dimensions that allow examination of justice across scales and over time (Figure A1).^70^

### A.1. Study setting

WHO WPR, a region that comprises 37 countries that are very different in terms of their size, economic affluence, governance structures and strength of healthcare delivery system.^71^ Some countries in the region are also known to face challenges of food security, while others are seeing the emergence of intensified food production practices.^72^ Finally, the region has had multiple infectious disease outbreaks and faces a significant threat from AMR. These factors informed my choice of WPR as the region for my analysis.^73^

### A.2. Study design and methodology

The study started with a broad review of purposively selected literature on Just Transitions, and Just Transitions in the context of climate change and labour movements to become familiar with the approach. Following this, the legal geography “JUST” framework was used to review global and regional WHO documents that guide actions in AMR. Through this review, key terms were identified and sense-checked with experts. Thereafter, NAPs were selected, and content analysis of eligible NAPs was conducted using the selected key terms as a guiding framework for data extraction and analysis.

### A.3. Identification and selection of key terms

*The Global Action Plan (2016), the Agenda for Antimicrobial Resistance in the Western Pacific Region (2015), the WHO six-point policy package to combat AMR (2011), and the WHO Global Strategy for Containment of Antimicrobial Resistance (2001)* were reviewed using the “JUST” framework to draft a list of key terms related to justice. The terms were categorised into Distributive, Restorative and Procedural Justice, and Time and Space components. An additional category was created that comprised words that are commonly used to describe justice and are relevant to all other categories, and was named “Synonyms of Justice.” For example, the word “equal” goes with equal representation for procedural justice, it also goes with equal distribution of cost in distributive justice and also with equal restoration in restorative justice.

The selected key terms and categories were then presented to experts in human health, public health, law, ethics, and community engagement to sense-check the applicability of these terms to the document review and analysis. New terms and synonyms identified during the review of NAPs were added to the list of key search terms, making the review of NAPs an iterative process using both deductive and inductive approaches.

### A.4. Identification and selection of NAPs for review

The WHO website has compiled and regularly updates publicly available NAPs of all the countries across different WHO regions of the world.^74^ NAPs for the Western Pacific Region available on the WHO website on May 7, 2023 were downloaded. NAPs were screened and duplicate versions and NAPs not in the English language were removed. The most recent version of the NAP that was available in English for each country was selected and included for review. Figure A2 gives a schematic diagram of the methodology.

### A.5. Analysis

The analysis started with a process of familiarisation with the NAPs, followed by a review of the overall goals/vision of the document. Following this, analysis was conducted in two distinct phases. Phase one involved measuring the frequency of identified key terms across the different NAPs using a keyword search in R software. The frequency of the search terms was aggregated according to the different components of the framework and reported per category rather than individual words to make a more meaningful interpretation.

In phase two, qualitative content analysis was performed, which involved careful reading of the NAPs and extraction of relevant sections where key terms were referred to. For this, a data extraction template was set up on Microsoft Excel in accordance with the categories in the JUST framework. The analysis was first done country-wise, and then inter-country comparisons were made according to the nature of governance systems and Gross National Income (GNI) per capita.^75^
